# 410. Preliminary results of 20-valent pneumococcal conjugate vaccine (PCV20) monitoring in children through 24 months of age in the Vaccine Safety Datalink

**DOI:** 10.1093/ofid/ofaf695.017

**Published:** 2026-01-11

**Authors:** TatYana Kenigsberg, Elizabeth Quincer, Lily Wang, Joshua T B Williams, Judith Maro, Robyn Kaiser, Jill Inderstrodt, Jason Glanz, Tierra Oseji, Lisa Jackson, Maria Sundaram, Nicola P Klein, Holly C Groom, Teresa Schmidt, Lei Qian, Eric Weintraub

**Affiliations:** Centers for Disease Control and Prevention, Atlanta, Georgia; Centers for Disease Control and Prevention, Atlanta, Georgia; Centers for Disease Control and Prevention, Atlanta, Georgia; Denver Health, Denver, Colorado; Harvard Pilgrim Health Care Institute, Boston, Massachusetts; HealthPartners Institute, Minneapolis, Minnesota; Indiana University, Indianapolis, Indiana; Kaiser Permanente Colorado, Denver, Colorado; Kaiser Permanente Mid-Atlantic States, DC, District of Columbia; Kaiser Permanente Washington Health Research Institute, Seattle, WA; Marshfield Clinic Research Institute, Marshfield, Wisconsin; Division of Research Kaiser Permanente Vaccine Study Center, Oakland, California; Kaiser Permanente Center for Health Research, Portland, Oregon; OCHIN, Portland, Oregon; Kaiser Permanente Southern California, Pasadena, California; Centers for Disease Control and Prevention, Atlanta, Georgia

## Abstract

**Background:**

In 2010, a 13-valent pneumococcal conjugate vaccine (PCV13) 4-dose series was licensed and approved for routine vaccination of children. In June 2023, the 20-valent pneumococcal conjugate vaccine (PCV20) 4-dose series was licensed and approved as an alternative for routine vaccination of children. Using surveillance data from the Vaccine Safety Datalink (VSD), we assessed post-licensure safety of the new PCV20 compared to a historical PCV13 control group. The VSD is a collaboration between the Centers for Disease Control and Prevention and 13 healthcare organizations that share electronic health record data and/or provide subject-matter expertise for vaccine safety surveillance and research studies.

**Methods:**

In February 2025, weekly sequential monitoring was initiated to estimate the rate ratio of 10 pre-specified adverse events (i.e., outcomes) following vaccination with PCV20 between June 2023-June 2025, compared to vaccination with historical PCV13 between June 2021-June 2023 in children aged 2, 4, 6, and 12-15 months of age. Analyses were conducted by PCV dose number and age group at vaccination. Within each subgroup, we used Poisson regression models with exact inference, stratified by age, sex, quarter, VSD site, and race and ethnicity.

**Results:**

Among 259,337 PCV20 doses and 549,321 PCV13 doses administer as of May 2025, there were no observed statistically significant signals for nine of the 10 outcomes monitored. We identified a statistically significant finding for urticaria/angioedema (ANGIO) following PCV20 dose 4 receipt (n=19,021 doses), when compared to PCV13 dose 4 receipt (n=80,960 doses) (rate ratio: 4.14; 95% CI: 1.19-16.48; *p*-value: 0.0251). Angioneurotic edema and vaccination-site urticaria reactions have been spontaneously reported during post approval use of PCV13 and may also be seen in post marketing experience with PCV20.

**Conclusion:**

During surveillance of the newly licensed PCV20 vaccine, the only statistically significant finding to date was a potential increased risk of ANGIO following PCV20 dose 4 receipt, compared to PCV13 dose 4. Medical record review to validate automated ANGIO cases is ongoing. Surveillance will conclude in November 2025, with plans for repeat analyses using chart confirmed cases.

**Disclosures:**

All Authors: No reported disclosures

